# Application of 2D Extension of Hjorth’s Descriptors to Distinguish Defined Groups of Bee Pollen Images

**DOI:** 10.3390/foods13193193

**Published:** 2024-10-08

**Authors:** Ewaryst Tkacz, Przemysław Rujna, Wojciech Więcławek, Bartosz Lewandowski, Barbara Mika, Szymon Sieciński

**Affiliations:** 1 Department of Clinical Engineering, Academy of Silesia, Rolna 43, 40-555 Katowice, Poland; szymon.siecinski@akademiaslaska.pl; 2 AI Technika Sp. z o.o., Włocławska 167, 87-100 Toruń, Poland; bartosz.lewandowski@aitechnika.com; 3 Department of Medical Informatics and Artificial Intelligence, Faculty of Biomedical Engineering, Silesian University of Technology, F.D. Roosevelta 40, 41-800 Zabrze, Poland; wojciech.wieclawek@polsl.pl (W.W.); barbara.mika@polsl.pl (B.M.); 4 Institute of Medical Informatics, University of Luebeck, Ratzeburger Allee 160, 23562 Lübeck, Germany

**Keywords:** bee pollen analysis, Hjorth’s descriptors, food product adulteration, pollen image parametrization, food product classification

## Abstract

Adulteration of food products is a serious problem in the current economy. Honey has become the third most counterfeit food product in the world and requires effective authentication methods. This article presents a new approach to the differentiation of bee pollen, which can support the development of a methodology to test honey quality based on the analysis of bee pollen. The proposed method is built on applying the Hjorth descriptors—Activity, Mobility, and Complexity—known from electroencephalography (EEG) analysis, for 2D bee pollen images. The sources for extracting the bee pollen images were the photos of honey samples, which were taken using a digital camera with a resolution of 5 megapixels connected to the tube of an optical microscope. The honey samples used were prepared according to the Polish standard PN-88/A-77626 (related to the European standard CELEX-32001L0110-PL-TXT). The effectiveness of the proposed method was positively verified for three selected groups of bee pollen—*Brassica napus*, *Helianthus*, and *Phacelia*—containing 35 images. Statistical analysis confirms the ability of the Hjorth descriptors to differentiate the indicated bee pollen groups. Based on the results obtained, there is a significant difference between the bee pollen groups under consideration regarding Activity p<0.00001, Mobility p<0.0001, and Complexity p<0.00001.

## 1. Introduction

In the modern economy, honey has become subject to improper labeling and the resulting consequences of falsifying its properties [1,2]. Due to the prevalence of fake honey products, the authentication of honey has become an active area of technical testing regarding its quality. Among the many countries that play an important role in the production market for honey and products derived from honey, New Zealand and the EU countries are the leaders, taking into account the protection of the interests of consumers and producers [3,4]. In the context of the above statement, more and more advanced methods for representing tested products are being sought. Research is usually of an indirect nature; that is, a mepollendium is sought that will well represent the food product, while, at the same time, creating the possibility of using the most advanced information processing technologies regarding the tested feature or the entire product. In the case of analyzing a food product, such as honey, this entails properly prepared pollen images or carrying out the so-called pollen analysis regarding pollen images [5,6]. There is considerable research on the composition and the pharmacological bioactivity of bee pollen (BP), indicating its usefulness and safety [7]. Numerous studies have shown that BP has a rich and well-balanced composition, being able to be served as a human food and supplement. Furthermore, its rich bioactive compounds, especially polyphenols, provide a variety of biological and pharmacological activities [8]. BP-related risks usually come from external factors [9] or from improper storage and processing conditions [8].

Allergic reactions are rare, and BP is perceived as a safe product in most physiological situations, including childhood, older age, and disease recovery [10,11]. BP from different floral sources has been reported to have anesthetic, antiallergic, anti-androgen, anti-atherosclerotic, anticancer (anticarcinogenic and anti-mutagenic), anti-inflammatory, antimicrobial (antibacterial, antifungal, and antiviral), antioxidant, antiulcer, and immunostimulant activities [12]. In metabolic pathophysiology, it has been shown to have antiobesity, antidiabetic [13], hypocholesterolemic [14], and hepatoprotective [11] effects. In the digestive system, it has been shown to maintain [10], improve [11], and regulate [14] intestinal functions. In the cardiovascular system, it can reduce capillary fragility [12] and improve overall cardiovascular health [15]. Some authors reported that BP can contribute to the prevention of and positively impact some degenerative processes such as neurodegeneration [15], general aging [13,14], and cellular apoptosis [16], and it can promote the recovery from chronic diseases and possess chemo-preventive properties [3]. It has also been shown to improve skin health and reparation, including many valuable cosmetic qualities [13].

Due to its established nutritional use, an ISO Norm (ISO 24382:2023) has been published to standardize the quality of bee pollen as a food product [17]. This clear interest in BP is evidently based on a solid background of evidence originating from the ethnopharmacological heritage and increasingly collected experimental data. Although BP in its isolated pellet format may be relatively recent and has emerged with the elaboration of mechanical pollen traps, bee bread and plant pollen have been used since ancient times. Ancient Egyptians described pollen as a “life-giving dust” [18], and Greeks believed that pollen and honey gave youth to kings [19]. Pollen was used for cosmetic purposes in ancient China [20]. Ancient Egyptians, Greeks, Native Americans, Chinese, and Indians have been reported to have used BP for food and energy on long journeys, as well as for other health benefits [21]. Based on this nutritional and ethnomedicinal history, BP use has been spreading at a very rapid pace during the recent few years due to emerging scientific evidence and the extensive development of the dietary supplement market around the world. BP is widely commercialized as a standalone food and nutritional supplement that benefits from its rich and well-balanced composition. The global market is expected to reach around EUR 670 million in 2024 [22].

One currently used method of assessing honey’s authenticity is melissopalynology. This method improves the accuracy of the analysis, both for identifying pollen types and for the precision of the respective concentration values [23]. Unfortunately, the protocol is carried out in a chemical laboratory setting, so the method is time-consuming, uncomfortable, and expensive. It seems reasonable to limit the applicability of lemon balm by searching for methods that would perform an initial selection of the cases studied. These methods aim to answer the following question: does a given case require such an expensive but detailed analysis, or can it be omitted? This gap is filled by the solution proposed in this paper. Therefore, we introduce a novel approach to the preliminary pollen pre-selection using Hjorth’s descriptors [5,6] to create a tool for a relatively simple and reliable classification that distinguishes counterfeit products that lack appropriate metrological characteristics.

The structure of this paper is as follows: In Section 2, we introduce the database of photographed honey samples in (Section 2.1) and the data processing pipeline consisting of data set preparation, normalization and filtration, feature extraction and selection using Hjort descriptors, and classification (Section 2.2). Section 3 presents the results. The summary of this study is presented in Section 4, and the conclusions are outlined in Section 5.

## 2. Materials and Methods

### 2.1. Materials

#### 2.1.1. Image Acquisition Procedure

Honey samples (AI Technika Sp. z o.o., Toruń, Poland) used to obtain bee pollen images analyzed in this paper were prepared according to the Polish standard PN-88/A-77626 [24] (related to European standard CELEX-32001L0110-PL-TXT) [25]. According to the pointed out procedure, the photographed honey sample was prepared as twice-centrifuged honey sediment taken from 10 g of honey. The centrifugation process lasted 10 min each time at a speed of 3000 rpm. Before the first centrifugation, the sample was topped with distilled water at 20 mL. After centrifugation, the liquid above the sediment was removed and refilled with distilled water at 20 mL. After the second centrifugation process, the liquid was decanted, and the remaining sediment was mixed and placed on a glass slide.

The photos were taken with a digital camera with a resolution of 5 megapixels, connected to the tube of an optical microscope. The parameter that defines the magnification of the lens was ×60.

Bee pollen was extracted from the photos using a convolutional neural network (CNN). The pre-trained ResNet50 model [26] was used as a base. The ResNet50 model was then trained based on the prepared pollen masks, which enabled it to effectively extract pollen images from honey samples.

ResNet-50 is a CNN architecture that belongs to the ResNet (Residual Networks) family, a series of models designed to address the challenges associated with training deep neural networks. Developed by researchers at Microsoft Research Asia, ResNet-50 is renowned for its depth and efficiency in image classification tasks. ResNet architectures come in various depths, such as ResNet-18, ResNet-32, etc., with ResNet-50 being a mid-sized variant. ResNet-50 was released in 2015 but remains a notable model in the history of image classification.

The primary problem ResNet solved was the degradation problem in deep neural networks. As networks become deeper, their accuracy saturates and then degrades rapidly. This degradation is not caused by overfitting, but rather by the difficulty of optimizing the training process.

ResNet solved this problem using Residual Blocks, which allow for the direct flow of information through the skip connections, mitigating the vanishing gradient problem. The choice of the ResNet-50 neural network was based solely on the simplicity of its implementation. There are no obstacles to using perhaps more advantageous solutions such as DenseNet [27] or HCGNet [28]. However, using the neural network in the context of this article is not the main problem addressed in this work. Hence, the network offering the most straightforward implementation, i.e., ResNet-50, was used.

#### 2.1.2. Database

**Figure 1:**
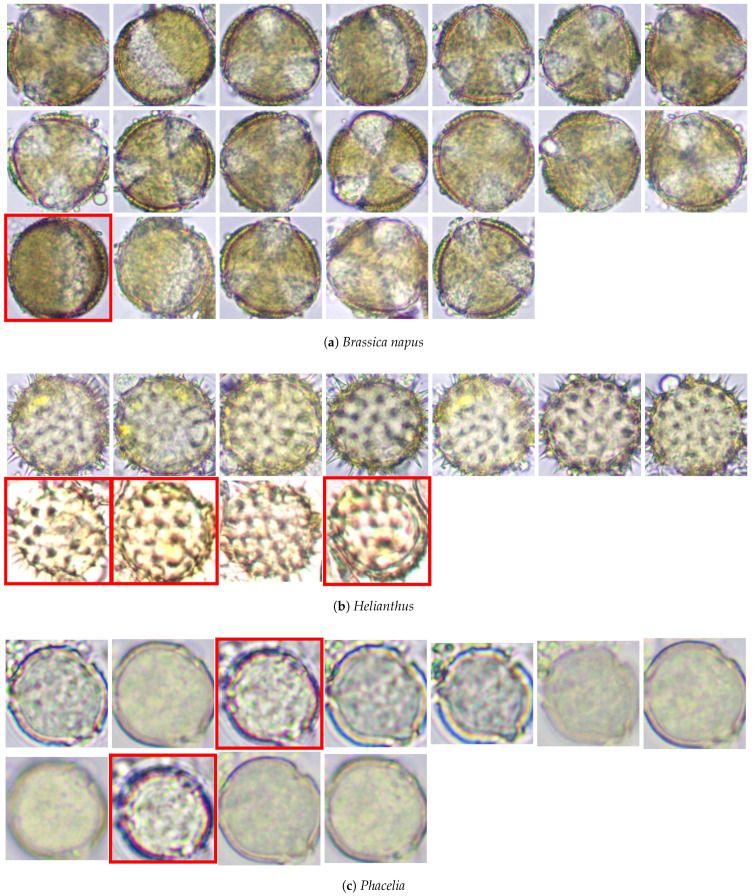
Bee pollen images divided into three groups (images marked in a red box were excluded from further analysis).

The testing database contained 41 color images divided into three groups: *Brassica napus* (19 images), *Helianthus* (11 images), and *Phacelia* (11 images), each with a resolution of 256 × 256 pixels and an 8-bit depth. The whole database is shown in Figure 1. In each of the three groups, some images were excluded (images marked in red boxes: *Brassica napus* (1 image), *Helianthus* (3 images), and *Phacelia* (2 images)) because they differed significantly from the remaining images in the group (the following image features were taken into account: overexposure, blur, and graininess).

Finally, 35 images (18 images of *Brassica napus*, 8 images of *Helianthus*, and 9 images of *Phacelia*) were used for further analysis.

### 2.2. Methods

**Figure 2:**
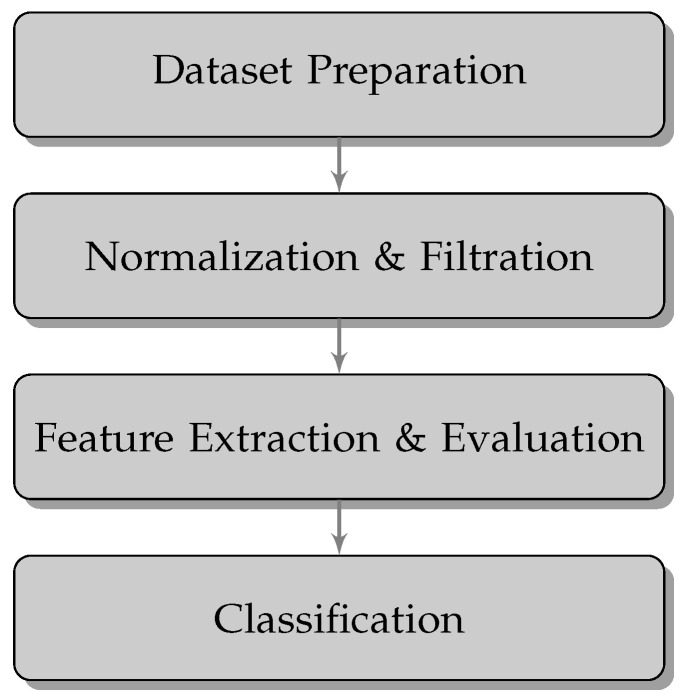
Workflow of the proposed methodology.

The proposed fully automatic bee pollen classification algorithm consists of several steps presented in Figure 2. After the preparation of the database procedure (described in Section 2.1), images were subjected to a Normalization and Filtration stage. Then, a Feature Extraction and Evaluation procedure was implemented. Finally, based on extracted features, the image Classification stage was performed.

#### 2.2.1. Data Preparation

Since the numerical analysis uses monochromatic images, the first step is to convert RGB color images (such as shown in Figure 1) to grayscale. A grayscale image may be defined as a two-dimensional function I(x,y), where *x* and *y* are spatial (plane) coordinates, and the amplitude of *I* in any pair of coordinates (x,y) is called the gray level of the image at that point. For simplicity, such an image will be denoted as *I*.

#### 2.2.2. Normalization and Filtration

The next stage after data preparation is intensity normalization and image filtration. The commonly used normalization approach, where the image intensity range is transformed into the range from 0 to 1, is Min–Max normalization or rescaling. It works according to the following formula:

(1) $$I\left(x,y\right)=\frac{I_{\mathrm{org}}-\min_{I_{\mathrm{org}}}}{\max_{I_{\mathrm{org}}}-\min_{I_{\mathrm{org}}}},$$

where $I_{\mathrm{org}}$ denotes the original image. This transformation does not affect the image itself.

Because the derivatives used later are very sensitive to noise, before they are determined, the normalized image is filtered using the average filter. Filter coefficients are calculated according to the average and standard deviation of the image.

#### 2.2.3. Feature Extraction and Evaluation

Further stages of the analysis will use image derivatives. As is commonly known, in the case of 2D signals, the derivative may be directional. However, the directionality of the derivatives in the developed approach is unimportant. For this reason, the gradient modules were determined using combined filters. In the case of the first derivative, the following formula was used:

(2) $$I^{\prime }\left(x,y\right)=\sqrt{{\left(\frac{\partial I}{\partial x}\right)}^{2}+{\left(\frac{\partial I}{\partial y}\right)}^{2}},$$

while, for the second derivative, the following one was used:

(3) $$I^{\prime \prime }\left(x,y\right)=\sqrt{{\left(\frac{\partial^{2}I}{\partial x^{2}}\right)}^{2}+{\left(\frac{\partial^{2}I}{\partial y^{2}}\right)}^{2}}.$$

For simplicity, the first and second image derivatives in any pair of coordinates (x,y) will be denoted as $I^{\prime }$ and $I^{\prime \prime }$, respectively.

**Figure 3:**
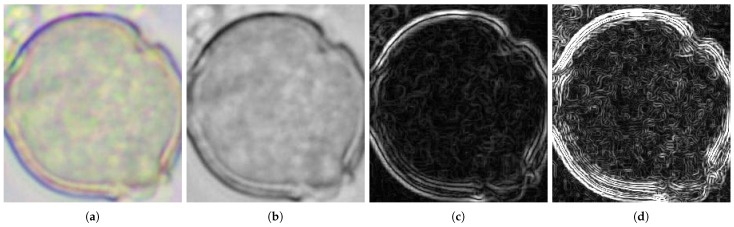
Single data set: (**a**) original color image (from Phacelia group), (**b**) original grayscale image *I*, (**c**) first derivative $I^{\prime }$ of the image, and (**d**) second derivative $I^{\prime \prime }$ of the image.

The above image preparation and derivative calculation stages are illustrated in Figure 3. The images presented in Figure 3b–d will be used for further analysis.

##### Hjorth’s Descriptors

The next step after image preparation, normalization, and filtering is feature extraction and evaluation. In the current submission, Hjorth’s descriptors were used to extract image features.

The information processing method that uses Hjorth’s descriptors is a little over 50 years old. In 1970 [5], the Swedish researcher Bo Hjorth proposed the construction of three descriptors—Activity, Mobility, and Complexity—to extract classification features and effectively evaluate EEG signals.

The research using Hjorth’s descriptors is based on a specific procedure that is relatively typical in terms of recognition and classification issues (see Figure 2).

The EEG signal is, as we know, a one-dimensional signal. Hence, an important novelty in the developed analysis methodology is an attempt to apply the above-mentioned descriptors in order to create the possibility of their application to two-dimensional signals, such as the images of bee pollen being analyzed.

The definition of Hjorth’s descriptors is derived from the frequency domain. The spectral moment of order *n* is defined as

(4) $$m_{n}=\int_{-\infty }^{+\infty }\omega^{n}\cdot S\left(\omega \right)\;d\omega ,$$

where $S\left(\omega \right)=J\left(\omega \right)\cdot J^{*}\left(\omega \right)$ is the power spectrum function. This function is computed as the product of the Fourier transform of the signal I(t) described as J(ω) and its complex conjugate $J^{*}\left(\omega \right)$. The spectral moment of order 0 (zero) $m_{0}$ represents the total power of the signal in the frequency domain, as follows:

(5) $$m_{0}=\int_{-\infty }^{+\infty }S\left(\omega \right)\;d\omega .$$

According to Parseval’s theorem [29], the mean power in the time domain is equal to the total power of the signal in the frequency domain, as follows:

(6) $$\mathrm{Activity}=m_{0}.$$

This spectral moment of order 0 is the first Hjorth’s descriptor called Activity [30]. The Activity of a signal is defined as the variance of the signal amplitude:

(7) $$\mathrm{Activity}=\sigma_{I}^{2}.$$

The dimension of the Activity parameter is the square value of the considered signal. It is worth noting that the variance of a zero-mean signal is equal to its mean power in the time domain:

(8) $$\sigma_{I}^{2}=\lim_{T\to \infty }\frac{1}{T}\int_{0}^{T}I^{2}\left(t\right)\;dt.$$

By analogy, the spectral moment of order 2 represents the average frequency of the signal:

(9) $$m_{2}=\sqrt{\frac{\int_{-\infty }^{+\infty }\omega^{2}\cdot S\left(\omega \right)\;d\omega }{\int_{-\infty }^{+\infty }S\left(\omega \right)\;d\omega }}$$

which is known as the second Hjorth’s descriptor called Mobility:

(10) $$\mathrm{Mobility}=\frac{\sigma_{I^{\prime }}}{\sigma_{I}}.$$

Finally, the spectral moment of order 4 represents the bandwidth of the following signal:

(11) $$m_{4}=\sqrt{\frac{\int_{-\infty }^{+\infty }\omega^{4}\cdot S\left(\omega \right)\;d\omega }{\int_{-\infty }^{+\infty }\omega^{2}\cdot S\left(\omega \right)\;d\omega }-\frac{\int_{-\infty }^{+\infty }\omega^{2}\cdot S\left(\omega \right)\;d\omega }{\int_{-\infty }^{+\infty }S\left(\omega \right)\;d\omega }}$$

which is the third Hjorth’s descriptor called Complexity:

(12) $$\mathrm{Complexity}=\frac{\frac{\sigma_{I^{\prime \prime }}}{\sigma_{I^{\prime }}}}{\frac{\sigma_{I^{\prime }}}{\sigma_{I}}}.$$

#### 2.2.4. Implementation

The fully automated algorithm was implemented in MATLAB (9.13.0.2049777, R2022b) and verified based on a desktop computer with AMD Ryzen 9 3900 12-core CPU at 3.1 GHz, 32 GB of RAM, and a Windows-10 64-bit operating system. The full-time analysis of all images (with statistical computations) was 12.315458 s.

#### 2.2.5. Statistical Approach to Bee Pollen Type Differentiation

Statistical analysis was performed to test the potential of Hjorth’s descriptors to discriminate between the different groups of bee pollen (*Brassica napus*, *Helianthus*, and *Phacelia*). The nonparametric Kruskal–Wallis ANOVA test and multiple comparison post hoc tests were applied for the Hjorth descriptors (i.e., Activity, Mobility, and Complexity) representing the bee pollen groups.

## 3. Results

**Figure 4:**
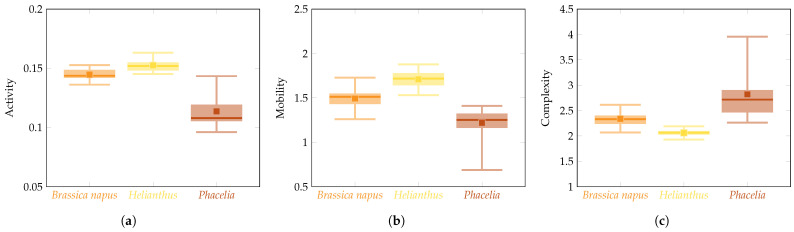
Box plots for Hjorth’s descriptors—(**a**) Activity, (**b**) Mobility, (**c**) Complexity—determined for three bee pollen groups: *Brassica napus*, *Helianthus*, and *Phacelia*. The box is drawn from the first to the third quartiles, with a horizontal line drawn inside to denote the median and a dot to present the mean. The whiskers indicate the minimum and maximum of a descriptor’s value for the group.

Figure 4 presents the box plots of the Hjorth descriptors, i.e., Activity, Mobility, and Complexity, calculated based on the bee pollen images, according to the procedure described above for the bee pollen groups comprising *Brassica napus*, *Helianthus*, and *Phacelia*.

The statistical approach mentioned in Section 2.2.5 was applied. In the first step of statistical analysis, the assumptions of a simple ANOVA test were verified. Due to the lack of a normal distribution and homogeneity of variance, confirmed by the Shapiro–Wilk test and the Levene test (Table 1 and Table 2), respectively, as well as due to the small size of the samples, the nonparametric Kruskal–Wallis ANOVA test (based on ranks instead of measurements) was applied for the Hjorth descriptors (i.e., Activity, Mobility, and Complexity) representing the bee pollen groups.

Kruskal–Wallis ANOVA test assesses whether n independent samples come from the same population (distribution) or the population with the same median (null hypothesis). According to the results summarized in Table 3, there is a significant difference between the bee pollen groups under consideration regarding Activity p<0.00001, Mobility p<0.0001, and Complexity p<0.00001. Based on the results presented, it can be stated that Activity, Mobility, and Complexity have a significant statistical impact on the affiliation of bee pollen to a specific group (*Brassica napus*, *Helianthus*, and *Phacelia*).

Multiple comparison post hoc tests were performed to answer the question of which of the compared groups was responsible for rejecting the null hypothesis, that is, to indicate which two pollen groups differed significantly. In line with the result obtained (Table 4, Figure 4) for Activity, significant differences were pointed out between the groups *Brassica napus* and *Phacelia*, as well as *Helianthus* and *Phacelia*; for Mobility, between the groups *Brassica napus* and *Helianthus*, as well as *Helianthus* and *Phacelia*; and, for Complexity, between the groups *Brassica napus* and *Helianthus*, as well as *Helianthus* and *Phacelia*.

**Figure 5:**
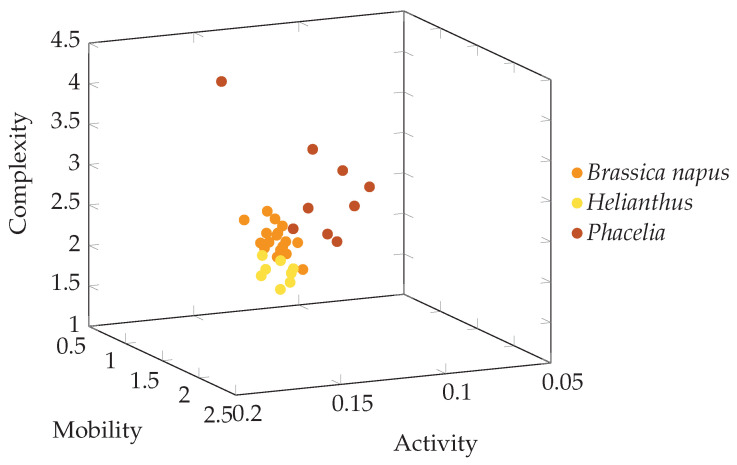
Three-dimensional visualization of analyzed image database of bee pollen three groups represented by points with coordinates of Hjorth’s descriptors (Activity, Mobility, Complexity).

The separation of individual image groups based on the Hjorth descriptors is also visible in Figure 5.

## 4. Discussion

This paper presents a novel approach to using the Hjorth descriptor method, which has been known for many years, originally developed for the analysis of one-dimensional electroencephalographic signals in the field of analysis of two-dimensional signals, such as bee pollen images. A detailed procedure for acquiring the images mentioned above, which became the basis for the analysis of their discrimination, was described. Therefore, a successful attempt was made to use image parameterization using Hjorth’s descriptors to construct an initial classifier of bee pollen images.

It should be clearly emphasized here that the primary goal for which such an attempt was made was to introduce some order into the honey production and distribution market, as there are rather chaotic and sporadic attempts to use modern digital tools to make honey production an important element of food product management.

In this paper, intensive statistical analyses were performed after a detailed description of the tool and the subject of this study, leading to interesting conclusions. Using the descriptors Activity, Mobility, and Complexity, it was possible to effectively distinguish the three groups of bee pollen studied. Therefore, appropriate tests of normality of the distribution of the parameters studied (Shapiro–Wilk test) and homogeneity of variance tests (Levene test) were performed. In turn, the rank of ANOVA (Kruskal–Wallis test) clearly indicated that the listed descriptors in the post hoc test clearly allow us to distinguish between the groups of bee pollen described above.

Therefore, to summarize this discussion, it can be stated that a successful attempt was made to parameterize bee pollen images using the Hjorth descriptors: Activity, Mobility, and Complexity. This first approach seems to create a reasonable hope of achieving the intended goal, which is a general and independent classifier of bee pollen for their general distinguishability and, subsequently, to check for possible frauds in the production of the final product.

## 5. Conclusions

The conclusions that emerge from this work can be summarized in two ways. First, in the opinion of the authors, a successful attempt was made to use a somewhat forgotten tool, which seems to be Hjorth’s descriptors, which were used for the first time over 50 years ago to parameterize two-dimensional signals, such as the images of bee pollen obtained in the manner described in the methods Section 2.2.5. Second, the use of the aforementioned descriptors, Activity, Mobility, and Complexity, to develop a methodology for distinguishing selected groups of bee pollen images effectively leads to the development of an effective classifier necessary for conducting qualitative analyses of food products such as honey.

The presented approach, although quite effective, has a few limitations. A fundamental one is the relatively small number of images analyzed in the three groups: *Brassica napus*, *Helianthus*, and *Phacelia*. The images of these selected bee pollen were obtained through a rather complicated technological process described in this paper. A study is underway to quantitatively enrich the database used for the authors’ analyses. Thus, as can be assumed, performing the analyses described in this article will definitely enable the method used here to gain credibility and allow it to be more representative. The authors of this paper set this as another goal aimed at developing an effective classification tool.

## Figures and Tables

**Table 1:** The smallest level of significance (*p*-value) for which the calculated value of testing statistic leads to rejection of the null hypothesis of normality distribution and homogeneity of variance for Shapiro–Wilk and Levene tests, respectively, for Hjorth’s descriptors: Activity, Mobility, and Complexity.

Hjorth Descriptors Variable	Normal Distribution Shapiro–Wilk Test *p*-Value	Homogeneity of Variance Levene Test *p*-Value
Activity	0.000177	0.001101
Mobility	0.017085	0.031298
Complexity	0.000026	0.000900

*p*-value (the level of probability *p*), the smallest level of significance for which the calculated value of testing statistic leads to rejection of null hypothesis.

**Table 2:** The exact results for the Levene test performed for the variance homogeneity of Hjorth’s descriptors (i.e., Activity, Mobility, and Complexity) to check the assumption of the simple ANOVA method of variance analysis.

Statistical Test	Hjorth Descriptors	Effect			Error			F	*p*
		SS	df	MS	SS*	df*	MS*		
Levene	Activity	0.000395	2	0.000197	0.000744	32	0.000023	8.491422	0.001101
	Mobility	0.079244	2	0.039622	0.327807	32	0.010244	3.867815	0.031298
	Complexity	0.563294	2	0.281647	1.023957	32	0.031999	8.801832	0.000900

SS—sum of squares between groups; df—number of degrees of freedom between groups; MS—mean squares between groups; SS*—within-group sum of squares (residual); df*—number of intra-group degrees of freedom (residual); MS*—mean number of squares within groups; F—F-test value (Fisher–Snedecor); *p*-value.

**Table 3:** The results for the ANOVA rank Kruskal–Wallis test performed for Hjorth’s descriptors (i.e., Activity, Mobility, and Complexity) obtained for three bee pollen groups: *Brassica napus*, *Helianthus*, and *Phacelia*.

Hjorth Descriptors	Group	Group Size	Rank Sum	Rank Average	Kruskal–Wallis	
					Test Value	*p*-Value
Activity	*Brassica napus*	18	348	19.333	21.478	0.00001
	*Helianthus*	8	229	28.625		
	*Phacelia*	9	53	5.888		
Mobility	*Brassica napus*	18	310	17.222	19.004	0.0001
	*Helianthus*	8	242	30.250		
	*Phacelia*	9	78	8.666		
Complexity	*Brassica napus*	18	339	18.833	20.944	0.00001
	*Helianthus*	8	41	5.125		
	*Phacelia*	9	250	27.777		

**Table 4:** The results (*p*-values) of multiple comparison post hoc tests performed for Hjorth’s descriptors—(**a**) Activity, (**b**) Mobility, and (**c**) Complexity—determined for three groups of bee pollen: *Brassica napus*, *Helianthus*, and *Phacelia*. Statistical significant differences between analyzed groups are marked with color (the color indicates pairs of bee pollen groups with significant statistical differences).

(**a**) Activity			
Group	*Brassica napus*	*Helianthus*	*Phacelia*
	RA = 19.333	RA = 28.625	RA = 5.888
*Brassica napus*		0.098529	0.003929
*Helianthus*	0.098529		0.000015
*Phacelia*	0.003929	0.000015	
(**b**) Mobility			
Group	*Brassica napus*	*Helianthus*	*Phacelia*
	RA = 17.222	RA = 30.250	RA = 8.666
*Brassica napus*		0.008313	0.122515
*Helianthus*	0.008313		0.000044
*Phacelia*	0.122515	0.000044	
(**c**) Complexity			
Group	*Brassica napus*	*Helianthus*	*Phacelia*
	RA = 18.833	RA = 5.125	RA = 27.777
*Brassica napus*		0.004926	0.097518
*Helianthus*	0.004926		0.000016
*Phacelia*	0.097518	0.000016	

RA—rank average.

## Data Availability

The original contributions presented in the study are included in the article, further inquiries can be directed to the corresponding authors.
